# Tissue Barrier-on-Chip: A Technology for Reproducible Practice in Drug Testing

**DOI:** 10.3390/pharmaceutics14071451

**Published:** 2022-07-12

**Authors:** Eugen V. Koch, Verena Ledwig, Sebastian Bendas, Stephan Reichl, Andreas Dietzel

**Affiliations:** 1 Institute of Microtechnology, TU Braunschweig, Alte Salzdahlumer Str. 203, 38124 Braunschweig, Germany; a.dietzel@tu-braunschweig.de; 2 Center of Pharmaceutical Engineering (PVZ), TU Braunschweig, Franz-Liszt-Straße 35 A, 38106 Braunschweig, Germany; v.ledwig@tu-braunschweig.de (V.L.); s.bendas@tu-braunschweig.de (S.B.); s.reichl@tu-braunschweig.de (S.R.); 3 Institute of Pharmaceutical Technology and Biopharmaceutics, TU Braunschweig, Mendelssohnstrasse 1, 38106 Braunschweig, Germany

**Keywords:** organ-on-chip, membrane, resealing technique, cell seeding, cell assay, tissue barrier, glass microsystem

## Abstract

One key application of organ-on-chip systems is the examination of drug transport and absorption through native cell barriers such the blood–brain barrier. To overcome previous hurdles related to the transferability of existing static cell cultivation protocols and polydimethylsiloxane (PDMS) as the construction material, a chip platform with key innovations for practical use in drug-permeation testing is presented. First, the design allows for the transfer of barrier-forming tissue into the microfluidic system after cells have been seeded on porous polymer or Si3N4 membranes. From this, we can follow highly reproducible models and cultivation protocols established for static drug testing, from coating the membrane to seeding the cells and cell analysis. Second, the perfusion system is a microscopable glass chip with two fluid compartments with transparent embedded electrodes separated by the membrane. The reversible closure in a clamping adapter requires only a very thin PDMS sealing with negligible liquid contact, thereby eliminating well-known disadvantages of PDMS, such as its limited usability in the quantitative measurements of hydrophobic drug molecule concentrations. Equipped with tissue transfer capabilities, perfusion chamber inertness and air bubble trapping, and supplemented with automated fluid control, the presented system is a promising platform for studying established in vitro models of tissue barriers under reproducible microfluidic perfusion conditions.

## 1. Introduction

Organ-on-chip systems have a high potential to improve in vitro studies for new pharmaceutical drug candidates and to accelerate the development of medication [1,2]. At a microscale level, the environment of cells can be precisely controlled, allowing the simulation of physiological conditions of particular cell barriers, such as the blood–brain barrier [3,4]. By mimicking these tissues, the permeation through the cell layer can be examined in detail [5,6]. To test the permeation properties of new drug candidates, the microfluidic systems only require a small volume of the drug solution. This in turn enables testing at an early preclinical stage, where the active ingredient is initially only available in small quantities. Although these microfluidic devices are easily accessible via academic research, the transfer of elaborated standard operation procedures, developed for static cell permeation assays of pharmaceutical drugs, remains challenging [7,8,9,10,11,12].

Compared to techniques in which the cells are cultivated statically on a polymer membrane, such as Transwell^®^ inserts, seeding the cells in a sealed microfluidic chip requires more sophisticated procedures [13,14,15,16]. During such procedures, a high fraction of cells is lost in the peripheral setup, and due to the typical parabolic flow velocity profile, an inhomogeneous distribution of cells occurs. Thus, even with the precise control of the cell-loaded fluid, the direct in-device seeding is far from being transferable to the laboratory routine. Furthermore, the precoating of integrated membranes is difficult, and a detailed examination of the tissue within a microfluidic chip is hampered by the lack of the applicability of staining protocols and by the typical working distances of the used microscope optics [7].

There are few known approaches that combine the benefits of microfluidic techniques with the reproducibility of standardized cell permeation assays that enable applications in large-scale pharmaceutical research. Known hybrid concepts are based on Transwell^®^ inserts, whereby the precultured cells on these inserts are transferred into the microfluidic chip for the experiment under flow conditions. Examples include the so-called DynaMiTES (Dynamic Micro Tissue Engineering System), which was presented earlier [17], and the multi-organ-on-chip platform developed and commercialized by TissUse GmbH [18,19]. In such a system, different barriers can be tested for their permeation properties, such as the cornea or the blood–brain barrier [14,15]. Although the hybrid concepts offer dynamic test conditions, the dependency on inserts limits the design freedom of channel geometries, and organotypic conditions can only be defined for a few in vitro models. Furthermore, when using cell culture inserts, only one side of the membrane is available for cell cultivation under dynamic conditions; thus, the flexibility to generate more complex cocultures is limited. Thus, a system is needed that is not only compatible with reproducible procedures for organotypic cultivation, but also allows the free design of similar microfluidic in vivo permeation testing. With regard to the blood–brain barrier, a channel system is desired that mimics the conditions in the brain capillaries with the appropriate shear stress to promote the formation of tight junctions and dense barriers.

Even though PDMS is widely used to form microfluidic devices by easy-to-realize soft lithographic fabrication [20], this material is associated with serious disadvantages, making the screening of pharmaceutical active ingredients and formulations doubtful [21,22]. The hydrophobic but porous surface of PDMS absorbs and desorbs small hydrophobic molecules and active substances. This distorts the determination of the permeated substance concentrations [23]. Furthermore, non-cross-linked oligomers, derived from the forming process of PDMS by mixing toxic oligomers with a cross-linker, diffuse slowly in the cell culture medium and influence cell growth [24]. In order to completely exclude the possible effects of these disadvantages, a high interest arose in either applying coatings for PDMS surface modification [25] or dispensing with PDMS [26] by using hydrogels, for example [27].

Here, we present an inert glass microfluidic perfusion chip with transparent electrodes that incorporates key innovations for pharmaceutical testing to overcome previous hurdles related to the transferability of existing static cell cultivation protocols, PDMS as a construction material, and long-term cell perfusion with outgassing media. The aim of the developments to be presented is to provide an organ-on-chip platform with practicable procedures for cell cultivation and tissue monitoring, so that a realistic test practice with high reproducibility in preclinical studies becomes possible.

## 2. Materials and Methods

### 2.1. Microfluidic Chip Design

**Figure 1:**
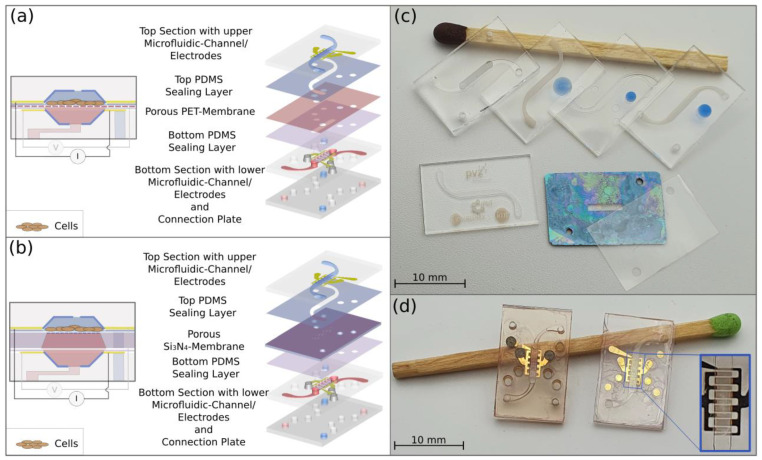
The microfluidic chip. (**a**) Schematic of the microfluidic chip from a cross-sectional view and an exploded view, illustrating the construction consisting of six layers: the top section, top sealing layer, porous polyethylene terephthalate (PET) membrane, bottom sealing layer and bottom section, made of two layers. (**b**) Schematic of the microfluidic chip with a porous Si_3_N_4_ membrane on a Si-chip from a cross-sectional view and an exploded view. (**c**) From upper left to right (lying on a match): bottom section with the lower microfluidic channel, the top section with the upper channel, the top section with the upper channel after annealing and placing a PDMS sealing layer according to the shift method, the top section with the upper channel with a PDMS sealing layer applied with the direct method (the hydrophobic PDMS sealing is visualized by the blue-colored water droplets in comparison to the spread droplet on the hydrophilic surface of the glass); from lower right to left: a porous PET membrane with through-holes, a Si-chip with a collagen-coated Si_3_N_4_ membrane in the center with through-holes and a glass chip after annealing, showing the S-shaped channel surrounded by the graphical feature. (**d**) Bottom section (left) with a lower microfluidic channel and interdigitated electrodes made of gold and ITO and the top part (right) with an upper channel covered by the sealing layer and the PET porous membrane, as well as a zoomed-in view of interdigitated electrodes.

The developed microfluidic chip, as illustrated in Figure 1a, consists of six layers and comprises compartments in the top section and in the bottom section, which are divided horizontally by a nanoporous polymer membrane, as used in Transwell^®^ inserts. The top section of the chip features an upper S-shaped microfluidic channel with a depth of 100 µm and a width of 1 mm. The two-layer construction of the bottom section contains the lower straight microfluidic channel, which has a depth of 200 µm and which defines the area of permeation through the membrane, and two embedded junction channels as well as four through-holes for the inlets and outlets. On the membrane, separating the channels, endothelial or epithelial cells are supposed to grow under physiological conditions in the constant flow of the culture medium. In order to seal the chip and prevent leakage, two thin layers of hydrophobic PDMS with recesses corresponding to the microfluidic layout are placed between the glass and membrane. The entire multilayered structure is then aligned and compressed in the microfluidic chip holder. As this sealing method allows for the integration of different materials, an ultrathin transparent membrane made of silicon nitride (Si_3_N_4_), as shown in Figure 1b, can also be integrated into the system. Compared to the polymer membranes, the ultrathin Si_3_N_4_ membrane with a thickness of 300 nm is more transparent, and in a coculture model, the intercellular communication through the membrane can be enhanced [6,28].

To monitor the barrier integrity of the cell layer, interdigitated microelectrodes made of transparent indium tin oxide (ITO) were embedded in the microfluidic channel (see Figure 1d). The electrodes enable the measurement of the transepithelial electrical resistance (TEER), which indicates barrier integrity. The interdigitated electrode design is beneficial for a uniform current density across the cellular monolayer, as recommended by Yeste, Illa et al. [29]. The tetrapolar (four-electrodes) configuration is accessible through the gold-coated contact pads next to the channel.

### 2.2. Fabrication of the Chip

The chip, as shown in Figure 1c, was fabricated from inert and optically clear borosilicate glass in a 4” wafer format (BOROFLOAT^®^ 33 with a thickness of 0.7 mm, Schott, Mainz, Germany) by a process adapted from Erfle et al. [30]. The structuring of the microfluidic channels and vias within glass wafers was realized using femtosecond laser (fs-laser) ablation using a laser workstation (Microstruct-C, 3D-Micromac, Chemnitz, Germany). The system was equipped with a Yb:KGW (ytterbium-doped potassium gadolinium tungstate) femtosecond laser source (Pharos, Light Conversion, Vilnius, Lithuania) emitting a fundamental wavelength of 1030 nm with a pulse duration of 212 fs. The deflection of the laser beam was controlled with a galvanometer scanner (Scanlab RTC5, Puchheim, Germany). The beam was then focused on the surface by an f-theta lens with a 100 mm focal length to a spot of around 20 µm in diameter exhibiting a Gaussian intensity distribution profile.

The layout of the contours for ablation were converted to a vector graphic consisting of a parallel-line pattern with a separation of 4 µm and an offset of 4 µm to the nominal edge. The parallel lines were orientated at 30°, 60°, 90° and 120° degrees to avoid periodic surface textures. For deep bulk ablation, the vector graphic was repeated in vertical distances of 50 µm, while the focus was adjusted. The laser’s writing speed was set to 1200 mm/s for all materials.

The microfluidic channels in glass were micromachined at pulse intensities of 12.2 µJ; for the vias, a higher pulse energy of 92.3 µJ was applied. After fs-laser structuring, the glass wafer was cleaned in an ultrasonic bath for 10 min in 50% ethanol in DI water and etched in an etchant made of 40% HF (hydrofluoric acid, Merck, Darmstadt, Germany) in DI water for 2 min to reduce the surface roughness of the channel. In contrast to the top section, the bottom section consists of two glass parts, one of which is laser-micromachined on both sides and the other contains only through-holes for the inlets and outlets. Both glass wafers were cleaned in a piranha etchant for 2 min and rinsed by a DI water jet before they were aligned and pressed together to create a pre-bond. The bond was then strengthened at 650 °C for 6 h under a weight of approx. 1 kg in a muffle furnace (VMK-135-S Sonder, Linn High Therm, Eschenfelden, Germany). In order to increase the transparency by reducing the surface roughness in the microfluidic channels, as shown in Figure 1c, the substrates were annealed at 750 °C for 1.5 h in the muffle furnace. Microelectrodes were fabricated starting with the magnetron sputtering of ITO (Laborsystem LS 440 S, Von Ardenne Anlagentechnik, Dresden, Germany) with a thickness of 270 nm on the entire surface of the glass wafer. A gold layer of 180 nm was sputtered on top of the ITO. Fs-laser ablation was used to selectively remove the gold and ITO layers from the glass, using a wavelength of 515 nm at pulse intensities of 1.26 µJ. Thereby, the shape of the microelectrodes was defined. In order to only remove the gold layer inside the microchannel, an adhesive tape (TESA, Hamburg, Germany) was pasted over the glass chip to cover all but the channel’s side and bottom walls, while a gold etchant (iodine/potassium iodide KI and DI water at a mixing ratio of 1/2/20) was introduced into the channel for 5 min and subsequently rinsed with DI water. For the bottom section, vias were realized by filling the hole with a conductive glue (Dualbond IC343, DELO, Windach, Germany), which was solidified at 80 °C for 3 h. Then, the glass wafer was placed in an ethanol bath to dissolve the adhesive layer and to remove the tape. Protruding hardened remains of conductive glue were removed by cutting them off with a sharp scalpel.

Commercially available PET membranes for cell cultivation (Sabeu GmbH, Northeim, Germany) with a thickness of 11 µm, a pore size of 0.4 µm and a pore density of 2.1 × 10^6^ pores/cm^2^ were cut into a chip format, and through-holes for accessing the upper microfluidic channel were drilled by fs-laser micromachining with pulse energies of 0.88 µJ at a wavelength of 515 nm.

Additionally, membranes made of Si_3_N_4_ were fabricated according to a process described by Ma et al. [31]. On top of an intermediate layer of silicon dioxide (SiO_2_) for film stress compensation, a layer of Si_3_N_4_ with a thickness of 300 nm was deposited on both sides of 360 µm-thick silicon wafers using a plasma-enhanced chemical-vapor-deposition process (PECVD) (310 PC, STS Surface Technology Systems, Yvonand, Switzerland). A square pattern defining the thin membrane area was transferred on the back side of the wafers by photolithography and the protective Si_3_N_4_ layer was opened by reactive etching with CF_4_ (200 sccm) and O_2_ (60 sccm) at 150 Watt (308 PC Barrel Etching System, STS Surface Technology Systems, Yvonand, Switzerland). By wet chemical etching in KOH at 80 °C for 8 h, the silicon was completely removed in the membrane area, leaving a thin membrane of Si_3_N_4_ in the square area surrounded by a more solid frame with the thickness of the full wafer. Through-holes in the Si-chip were made via micromachining using pulse energies of 20 µJ at a wavelength of 515 nm. In the future, nanopores could also be created in the membranes by high-resolution photolithography.

### 2.3. Fabrication of the Sealing Layer

**Figure 2:**
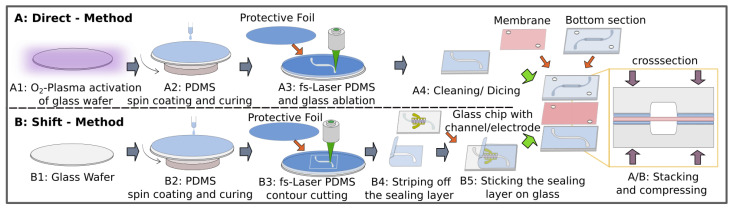
Two approaches for the application of the PDMS sealing layer. (**A**) Direct method: the glass-bonded thin PDMS sealing layer is structured in a single femtosecond laser (fs-laser) ablation process. (**B**) Shift method: the PDMS sealing layer is contoured using the fs-laser and is manually peeled off the substrate and placed on the processed microfluidic glass chip. As a last step, all layers are stacked and compressed in a microfluidic chip holder.

Thin PDMS sealing layers, as illustrated in Figure 2A (direct method), were fabricated using 184 silicone elastomer, which was supplied as a two-component kit (SYLGARD TDCC, Midland, MI, USA). After mixing both reagents together and degassing at 0.2 mbar in a vacuum chamber, the elastomer was spin-coated on 4ʺ glass wafers, which had been surface-treated with 50 W at 13.56 MHz for 5 min by oxygen plasma (Plasma Active Flecto 10, Plasma Technology, Herrenberg-Gueltstein, Germany). For curing, the wafers were placed on a hotplate for 1 h at 70 °C.

After covering the PDMS layer with a foil (Tecni-Tape, DISCO HI-TEC EUROPE, Kirchheim, Germany) to protect the surface from the deposition of debris particles, the femtosecond laser was used in combination with a harmonic generator to structure the PDMS layer and the glass substrate underneath by laser ablation at a wavelength of 515 nm with pulse intensities of 14 µJ. At the last step, the structured PDMS-coated glass wafer was diced and cleaned by rinsing with 50% ethanol in DI water.

As an alternative to the direct method, a shift method was also implemented, as shown in the bottom row in Figure 2B. In order to allow the sealing layer to be peeled off, the surface of the glass wafer was not activated by an oxygen plasma prior to spin coating and curing. The contour of the sealing layer was cut out by the fs-laser. Then, the sealing layer could be transferred with sufficient alignment to the glass chip, fabricated separately as described above. The alignment of the sealing layer could be facilitated by wetting the interface surfaces with ethanol.

The direct method is easy to apply, as the sealing layer remains on the glass, allowing very thin layers below 20 µm. A disadvantage of this method is that the microfluidic channel still has a relatively rough surface after micromachining, as shown in Figure 1c, which can interfere with an optical analysis of the cells. With the shift method, the glass substrate can still be processed for smoothening the channel walls by thermal annealing and for implementing electrodes before bonding. Using the shift approach, the sealing layer has to be thicker, around 50 µm. This makes the seal not only manually manageable but also reusable and, similar to conventional gaskets, allows the leak-tight sealing of materials that cannot be bonded with PDMS. To test different PDMS qualities, the volume-mixing ratios of the two components and the spinning speeds during coating were varied. Samples with PDMS-mixing ratios of 2:5 (28.57 wt%), 1:5 (16.67 wt%), 1:10 (9.09 wt%), 1:15 (6.25 wt%), 1:20 (4.76 wt%) and 1:25 (3.85 wt%) (curing agent to base elastomer, in weighted ratios) were molded in the forms of 5 cm in diameter and 5 mm in depth. The samples were cured at 70 °C for 72 h on a hotplate before the hardness was tested at 37.5 °C, in accordance with DIN ISO 7619-1 (2012-02), using a benchtop hardness tester (Bareiss, Oberdischingen, Germany). The measurements were recorded 3 s after complete indentation in order to mitigate the relaxation of the elastomer. For the thickness measurement, spin-coated PDMS was removed from the glass substrate in 500 µm × 500 µm squares by PDMS selective fs-laser ablation (wavelength 515 nm). Subsequently, debris was removed by cleaning the wafer in ethanol for 10 min in an ultrasonic bath. The topography across these squares was measured with a confocal 3D laser scanning microscope (VK-X260K, Keyence Deutschland, Neu-Isenburg, Germany), using 50× magnification optics.

### 2.4. Microfluidic Chip Holder

**Figure 3:**
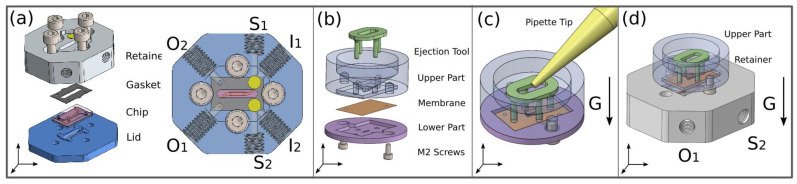
Microfluidic chip and transfer holder (**a**) Explosive view of the system holder (left), consisting of a retainer, a gasket, the chip and a lid. On the right, the assembled microfluidic setup with the assigned ports. (**b**) Explosive view of the assembled transfer holder as applied for seeding cells on the membrane, consisting of an ejection tool, the upper and lower part, a pair of M2 PEEK screws and a porous membrane for cell cultivation (left). (**c**) Illustration of the membrane coating and cell-seeding process by cell injection through a pipette. (**d**) Illustration of the transfer process of the cultivated membrane from the upper part of the transfer holder to the microfluidic chip. The upper part is coupled to the retainer, which is inverted during the transfer, and the membrane is fixed on the bottom section of the chip with the inserted ejection tool. The arrow marked with “G” indicates the direction of gravity.

Reversible sealing requires an adequate holder, which firstly enables the alignment of all five layers in the stack and secondly, compresses the stack to reliably seal the microfluidic chip. The holder, as schematically shown in Figure 3a, consists of a retainer (white part), in which the microfluidic chip is placed, and a lid (blue part), which compresses the system using four stainless-steel M4 screws. A gasket made of silicone with holes for the inlets and outlets of the microfluidic system is placed between the microsystem and the retainer.

**Figure 4:**
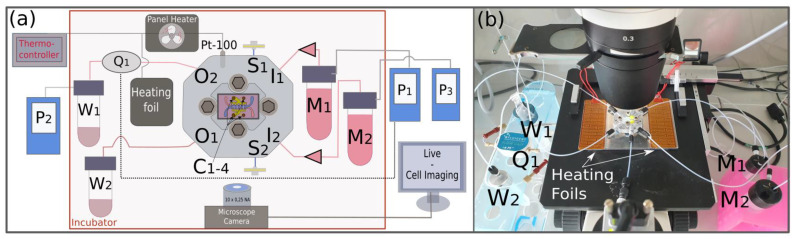
(**a**) Schematic view of the experimental setup for flow control showing the microsystem in the center of the retainer (with inlets I1 and I2, outlets O1 and O2, side ports S1 and S2 and contact pad holes C1–4), culture medium reservoirs (M1, M2), waste reservoirs (W1, W2), pneumatic controllers (P1, P2, P3), the flow-rate sensor (Q1) and sterile syringe filters connected to side ports S1 and S2, which are closed during the experiments. The main setup is positioned on top of an inverse microscope placed in a self-built incubator (red frame) using a thermocontroller coupled to a panel heater and heating foils and to a Pt-100 temperature sensor. (**b**) Actual image of the microsystem in the center surrounded by the peripheral flow control.

The retainer is made of transparent polycarbonate (Makrofol, Arla Plast, Borensberg, Sweden) by standard 2D-CNC milling and has six ports for inlets, outlets and side ports, allowing the release of entrapped air before the start of the experiment. The microsystem can be connected through the ports to the experimental periphery, shown in Figure 4. Additional openings vertically connected to the inlet channels are closed with air-permeable membranes of polytetrafluoroethylene (PTFE) (DAM-AD10, B+B Thermo-Technik, Donaueschingen, Germany) shown as small yellow circular areas in Figure 3, allowing the continuous release of air bubbles during experiments.

In order to have access to the contact pads of the microfluidic system for TEER measurement, four holes (C1–4) are located in the center, as shown in Figure 3.

An oval gap in the center of the retainer and the lid allows the analysis of the permeation region of the microfluidic chip by optical microscopy. The lid is made of aluminum and has four holes with threads to tighten the screws. As illustrated in Figure 3a, a raised structure on the lid in the form of the microfluidic design guides the force of the screws directly into the system and creates a uniform pressure on the layers. To prevent the canting and subsequent breaking of the glass microsystem, it is crucial to tighten the screws alternately and in steps, applying a torque wrench set to a maximum of 0.15 Nm.

### 2.5. Transfer Holder

One major advantage of the reversible chip-sealing technique is the possibility of the external seeding and immobilization of the cells on a porous membrane according to static cultivation protocols. To facilitate the transfer of the membrane to the chip, a transfer holder was designed, which accommodates membranes in a similar manner as in Transwell^®^ inserts.

As illustrated in Figure 3b, the upper part of the transfer holder has a similar design as a funnel-shaped pot with a small oval-shaped opening at the bottom at a size of 0.5 × 3.3 mm, which is slightly smaller than the cell cultivation area in the chip. Four additional round holes are placed around the opening to allow the membrane to be released evenly with the aid of an ejection tool. Its four pins can be inserted into the round holes to push the membrane. The lower part (pink part) has a similar hole pattern and a groove where the membrane (orange layer) is placed. Both parts and the membrane in the middle are pressed together by two M2 screws made of polyetheretherketone (PEEK). The coating of the membrane and subsequent seeding of the cells, as illustrated in Figure 3c, can be performed according to standardized static cell assay protocols by pipetting.

Once the lower part of the transfer holder is removed and the screws are screwed in again, the upper transfer holder with the attached membrane underneath and the retainer can be plugged together using the screws as guiders, and raised square structures at the bottom of the upper transfer holder facilitates the connection with the retainer. During this step, the membrane is fixed to the upper transfer holder by capillary forces caused by a thin liquid film of cell culture medium between these parts. In Figure 3d, the positioning of the transfer holder to the inverted retainer and the ejection tool during membrane transfer is illustrated. The membrane is evenly pushed into the retainer with the aid of the ejection tool, where the bottom section of the microfluidic chip, including the PDMS sealing layer, is already placed. In contrast to the upper and the lower part of the transfer holder, which are also made of polycarbonate via 2D CNC milling, the filigrane ejection tool is realized by 3D printing (AR-M2 material, Agilista 3200W, KEYENCE Germany, Neu-Isenburg, Germany).

After the top section of the microsystem with the PDMS sealing layer is placed on the membrane, the microfluidic chip holder can be turned back again and the lid can be tightened by the screws. The enclosed system can then be connected to the peripheral experimental setup. The membrane transfer process is shown in detail in the animation (Supplementary Video S1).

### 2.6. Cell Cultivation

All the membranes were first treated by an oxygen plasma for 90 s to improve the wettability for coating with a rat-tail collagen solution (1.5 mg/mL dissolved in ethanol and acetic acid, 0.05%). After the membranes were dried, they were inserted into a transfer holder and also inserted directly into the microfluidic chip in order to compare the two different cell-seeding methods. For all cell-related experiments, MDCK (Madin–Darby canine kidney) cells that were received from the European Collection of Authenticated Cell Cultures (ECACC) were used. The cells were cultivated in Eagles MEM culture medium (PAN Biotech, Aidenbach, Germany) containing 2.2 g/L NaHCO_3_, 10% (*v*/*v*) FBS (Merck, Darmstadt, Germany), 2 mM L-glutamine (Biochrom, Berlin, Germany), and 1% (*v/v*) antibiotic/antifungal mixture (Merck, Darmstadt, Germany). For the direct seeding method, which is based on the introduction of cell suspension into the channel and the sedimentation of the cells, as described by Akther et al. [25], volumes of 200 µL with a cell suspension of 2.5 × 10^6^ cells/mL were used.

For the transfer method experiments, a cell suspension of 1 × 10^6^ cells/mL was used to realize a cell-seeding density of 13,750 cells/mm^2^. Seeding densities of 900 cells/mm^2^, 4500 cells/mm^2^ and 13,750 cells/mm^2^ were applied for the reference measurements in Transwell^®^ inserts. After a confluent cell layer was developed, the membrane was transferred into the microfluidic chip by the procedure described above.

The MDCK were examined for viability by live–dead cell staining with a mixture of 0.02 mg/mL propidium iodide (Merck, Darmstadt, Germany), 0.005 mg/mL calcein-AM (Merck, Darmstadt, Germany) and 0.02 mg/mL bisbenzimide H 33342 trihydrochloride (Hoechst 33342) (Merck, Darmstadt, Germany) in Krebs–Ringer buffer (KRB). Cells were incubated with this solution for 30 min under light protection at 37 °C, followed by two washing steps with KRB [32].

In order to prove the biocompatibility of the installed materials, samples were brought together with MDCK cells. Samples of silicone rubber GP 60 (MVQ Silicones, Germany), double-sided adhesive tape (TESA, Hamburg, Germany), polycarbonate holder material, and PDMS, each with a diameter of 13 mm and varying thicknesses, were placed on the well bottom of a 12-well plate (Corning Costar, Corning, NY, USA). In parallel, MDCK cells were cultivated without any additional material as a negative control. A total of 50,000 MDCK cells were seeded per insert (Corning Costar, PET, 0.4 µm pore size). After five days of cultivation, an MTT assay was performed to determine the viability of the cells [33]. For the MTT assay, a 1:10 dilution of a 0.5 % MTT solution (Merck, Darmstadt, Germany) was prepared with the cell culture medium, and the cells were incubated at 37 °C in the incubator, whereby the tetrazolium dye MTT converted into the insoluble formazan only by the viable cells. This incubation medium was then replaced after 2 h by incubation at room temperature for 10 min with DMSO (Fisher Scientific, Waltham, MA, USA). The samples were then transferred to a 96-well plate (TPP, Switzerland) and the absorbance was measured with a microplate reader at 570 nm (PowerWave XS, BioTek, Winooski, VT, USA). All handlings were carried out in a clean bench (Fisher Scientific SAFE 2020, Fisher Scientific, Schwerte, Germany). The growth experiments in this study, except those in the chip, were carried out in an incubator at 37 °C with 5% CO_2_.

### 2.7. Experimental Setup

The microfluidic systems were tested in a setup with pressure control at both inlets and one outlet as schematically shown in Figure 4a. The inlets I1 and I2 of the chip holder (see Figure 4b) were connected to the cell growth medium reservoirs M1 and M2. The fluidic flow was realized by two pneumatic controllers P1 and P3 (MFCS-EZ, Fluigent, Le Kremlin-Bicêtre, France) supplied by carbogen gas (5% CO_2_ in O_2_). Growth medium that passed the microfluidic chip was collected in waste reservoirs W1 and W2. In order to control the flow rate in the upper channel, a flow-rate sensor Q1 (Flow Unit S, Fluigent, Le Kremlin-Bicêtre, France) was placed at the outlet of the system and linked to the pneumatic controller P1. The flow through the lower channel was controlled by pressure at the inlet without feedback. All the parts were connected via flangeless fittings made of Delrin (H&S P-307, IDEX, Lake Forest, IL, USA) via polytetrafluoroethylene (PTFE) tubes (ID = 0.750 mm, TechLab, Germany). Before the start of an experiment, any trapped air in the tubing connected to the inlets was released through the side ports S1 and S2. Those were connected to 3-cm-long tubings, which were closed with syringe filters (PTFE, 0.2 µm, Whatman, Maidstone, UK) after the air was removed. Prior to connecting the cell media reservoir to the setup, it was degassed at 20 mbar for 5 min to reduce the concentration of dissolved gas in the culture medium. The risk of air bubble formation inside the chip could be further decreased by rising the pressure in the upper channel by 15 mbar using the pneumatic controller P2. This way, air bubbles can also be pushed out of the system before entering the microsystem. Air bubbles in the microsystem can still exit via the air-permeable membrane at the inlet of the retainer. To measure the TEER, the gold-coated contact pads on the chip can be accessed through the four holes (C1–4) via a connector.

As indicated in Figure 4 by the red frame, the experimental setup was placed in a self-built incubator, made of an aluminum platform containing heating foils, a panel heater with a 250 W fan (SH 250L, Elmeko, Liebenscheid, Germany) and an integrated sensor (Pt-100) controlled by a thermocontroller (JUMO di eco, Jumo, Fulda, Germany) to set a temperature of around 36.5 °C. The platform was covered by a housing made of polymethylmethacrylat (PMMA). The incubator was placed on an inverted microscope (Primovert, Zeiss, Wetzlar, Germany) equipped with 4×, 10× and 20× plan achromatic objectives, and a microscope camera (PowerPack ace 3.2 MP, Basler, Ahrensburg, Germany), allowing the continuous monitoring of the cell growth by time-lapse recording. The incubator’s only function is to stabilize a temperature of 37 °C, and any other incubator could be used as well. A photo of the experimental setup shows the detailed setup (Supplementary Figure S2).

### 2.8. LIF Measurements

The absorption of substances and the sealing ability of the PDMS layer were examined by laser-induced fluorescence (LIF). The upper section of the microfluidic chip was fabricated according to the direct method procedure with mixing ratios of 1:20 (4.76 wt%), 1:15 (6.25 wt%), 1:10 (9.09 wt%), and 1:5 (67 wt%). At 2500 rpm, a sealing layer thickness of 25 µm was obtained. The sample was pressed against a glass blank with the outer dimensions of the bottom section and closed in the chip holder. The marker substance rhodamine B chloride LOT (type 610) (purchased via LaVision, Göttingen, Germany) was diluted in distilled water with a concentration of 0.12 mg/mL. Rhodamine B with a partition coefficient log P of around 1.95 can be considered as representative for many hydrophobic pharmaceutical substances. The dyed liquid was introduced into the microfluidic channel with rising pressures from 200 mbar to 1 bar in 200 mbar steps using the pneumatic controller, realizing a flow rate of approximately 1.2 mL/min (1 bar). At each step, the pressure was kept constant for around 2 min, leading to a total dwell time of around 10 min.

Prior to loading with dyed water and at the end of the experiment, the chip was filled with undyed DI water to measure the background fluorescence as well as the residual concentration in the PDMS.

The measurements were performed with a Stereo-Micro-PIV FlowMaster System (LaVision, Göttingen, Germany), comprising a Zeiss V20 stereomicroscope (Carl Zeiss, purchased via LaVision) with two Imager sCMOS double frame cameras (LaVision) and a Litron Bernoulli double-pulse Nd:YAG laser (532 nm, 100 mJ, Litron Lasers Inc., purchased via LaVision) coupled with an optical fiber to the setup. The system was synchronized using a programmable timing unit (PTU X) (LaVision). The recording and evaluation of the test were performed with Davis 10 software (LaVision).

### 2.9. Gold-Etching Leak Test

Furthermore, a self-developed leak test was applied. The top and bottom glass slides were annealed after fs-laser ablation to reduce channel roughness and then coated with a thin layer of chromium (10 nm) and gold (180 nm) by magnetron sputtering. The structured sealing layer was fabricated according to the shift method with a thickness of around 80 µm and placed on the gold-coated glass slides. After the complete system was assembled, a gold etchant (iodine/potassium iodide KI and DI water at a mixing ratio of 1/2/20) was pressed into the upper microfluidic channel for 5 min.

After flushing with DI water until the yellow-colored etchant was completely removed, the system was disassembled and optically inspected. As a reference, a leakage was forced by assembling the system with a fiber obstacle (70 µm) placed between the polymer membrane and the PDMS sealing layer.

### 2.10. TEER Measurements

The on-chip TEER measurements were performed using an EVOM2 (World Precision Instruments, Sarasota, FL, USA) at room temperature. The chip was connected to the device via a customized connector with spring pins connected to the gold pads of the microfluidic system. As an off-chip reference, a commercial EndOhm 12 G chamber (World Precision Instruments) was used.

## 3. Results and Discussion

### 3.1. Gas Trapping

**Figure 5:**
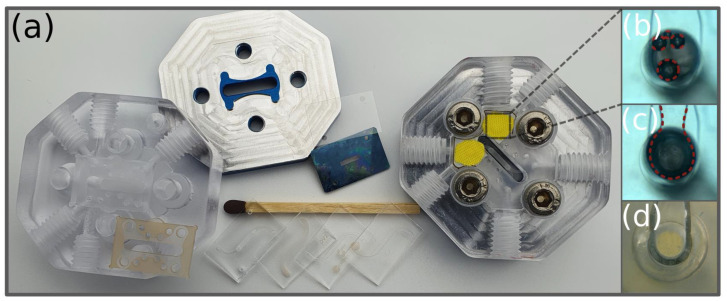
Microfluidic retainer. (**a**) Photograph of the complete microfluidic setup including chip and retainer elements. (**b**) Air bubble from the outgassing medium trapped in the center of the inlet port in the retainer, which is not equipped with a phase filter membrane. (**c**) Growing air bubble entering the upper microfluidic channel (highlighted by the red dashed line), 15 min after filling the chip with the culture medium. (**d**) Inlet port in a retainer equipped with a phase filter membrane to remove air before entering the microchannel.

Inspired by the work of Zheng et al. [34], the chip holder was equipped with side ports S1 and S2 (Figure 4) through which trapped air from the peripheral tubing could be evacuated. In addition, the inlets were in connection with vertical channels, which were closed with an air-permeable phase filter. Without these gas-escape features, small air bubbles typically formed at the inlet port directly after the continuous culture media supply was started, as shown in Figure 5b. During cultivation, the gas bubbles grew and entered the upper microfluidic channel after 15 min (Figure 5c). While passing through the channel, the air bubbles damaged the cell layer, not only by locally drying it out and undersupplying it with culture medium, but also by pulling and tearing off individual cells, as can be seen in a recorded video (Supplementary Video S2), which is supplied in the supplementary data. With the installation of gas-permeable membranes, bubbles generated during the cultivation phase could reach the membranes (Figure 5d) to escape the system. This way, long-term, stable on-chip cultivation became possible, enabling dynamic cell cultivation for at least 72 h.

### 3.2. Material Cell Compatibility Tests

In order to verify that the materials used in the chip and the periphery (as shown in Figure 5a) had no negative influence on cells, a viability test was carried out. The silicone gasket was applied at the interface between the chip and the retainer. During cultivation, only a small area of the silicone rubber came in contact with the culture media. As the transfer holder and the retainer were made of PC, the cells stayed in direct contact with the material prior to the transfer. Afterwards, only the culture media passed through the channel inside the retainer. The structured PDMS sealing layer stayed throughout the experiment in the immediate vicinity of the cells. The double-sided TESA film was used to attach the air-permeable phase filter on the system retainer, and therefore was not in contact with the cells. After 5 days of cultivation of the cells with the tested materials, no significant negative effect was observed. By means of an MTT test, a cell viability of above 90% was measured (Supplementary Figure S1), indicating sufficient biocompatibility. Due to excellent biocompatibility, the adhesive film could also be applied as an alternative sealing option instead of the PDMS sealing layer, as described for double-sided adhesive tapes by Kratz et al. [35].

### 3.3. Sealing-Layer Parameters

The PDMS sealing layer’s thickness could be adjusted between 17 µm (at 4000 rpm of the spin coater) and 58 µm (at 1000 rpm,). The entire wetted inner surface area of the upper microfluidic channel was around 13.03 mm^2^. The area spanning a 17-µm-thick sealing layer, which came in contact with the fluid, made up only 2% of it. Even with a sealing layer thickness of 58 µm, the PDMS contact area would still be less than 7%.

**Figure 6:**
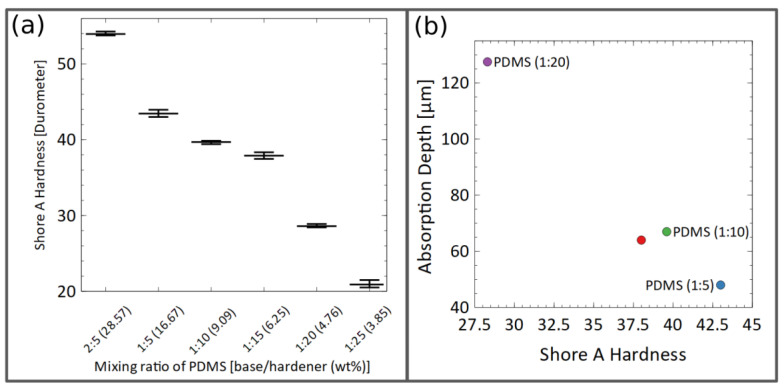
(**a**) Dependence of the Shore durometer hardness from the mixing ratio of PDMS, measured at 37 °C. (**b**) Correlation of the durometer hardness with the absorption depth of rhodamine B in the sealing layer.

As can be seen in Figure 6a, the durometer hardness declined nearly linearly with an increasing amount of curing agent to the elastomer within a range of 21.2 Shore A (PDMS 1:25), which is comparable to chewing gum, to 54 Shore A (PDMS 2:5), corresponding to a car tire. Between the PDMS compositions of 1:10 and 1:15, the durometer hardness was similar. At a higher hardness, the material showed a stronger resistance to indentation. Thus, protruding structures or rough surfaces could not be sealed as easily. However, soft seals also deformed more easily at higher working pressures and lacked form stability. The measured hardness for the PDMS composition of 1:10 is comparable to the results published by Johnston et al., who analyzed the dependency of the durometer hardness of PDMS (1:10) on the curing temperature of PDMS [36].

### 3.4. LIF Measurements

**Figure 7:**
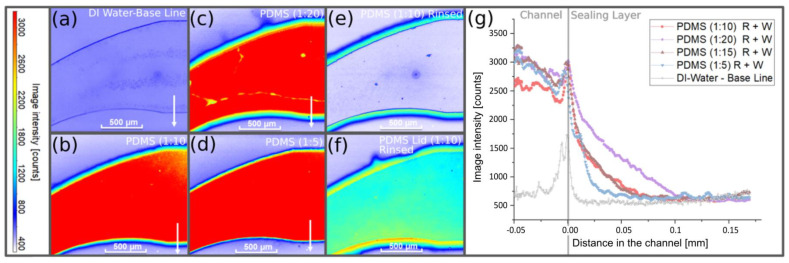
Fluorescence images and fluorescence intensity distributions in the sealing layer observed within the microfluidic upper channel filled with DI water and rhodamine B dissolved in DI water (R+W) at 1 bar. (**a**) Channel with a PDMS sealing layer filled with DI water. (**b**) Channel with a PDMS sealing layer (1:10) (9.09 wt%) filled with dyed liquid. (**c**) Channel with a PDMS sealing layer (1:20) (4.76 wt%) filled with dyed liquid. (**d**) Channel with a PDMS sealing layer (1:5) (16.67 wt%) filled with dyed liquid. (**e**) Channel with a PDMS sealing layer (1:10) (9.09 wt%) after rinsing the channel with 1 mL DI water. (**f**) Channel with a sealing layer (PDMS 1:10) (9.09 wt%) enclosed by a glass lid coated with PDMS (PDMS 1:10) (9.09 wt%), after rinsing the channel with 1 mL DI water. (**g**) Correlation of the absorbed rhodamine B with the sealing layer by applying different PDMS mixing ratios.

Figure 7 shows a section of the microfluidic upper channel at the inlet curvature. The obtained fluorescence images are corrected by the background intensity, so that light intensities correspond to the dye concentration in the liquid. The softer the PDMS sealing layers are, the progressively more blurred the contour in Figure 7 appears to be, as the dye diffuses more deeply into the PDMS. For a PDMS ratio of 1:20, radial leaking stripes also become visible.

For a more quantitative analysis, intensity line scans were extracted at positions indicated by the white arrows (Figure 7a–d). The scans were smoothened using a second-order moving average filter. The zero position in the line scans corresponded to the channel edge (transition from the microfluidic channel to the sealing layer) where signal overshoots due to light scattering at the roughened channel walls were already observed in chips filled only with DI water. The intensity profiles showed that the diffusion depth and the amount of absorbed rhodamine B (area underneath the intensity curve) increases when the PDMS was softer (fabricated with a higher elastomer concentration).

Although the absorption properties of the PDMS ratios of 1:10 and 1:15 were nearly identical, the increase in the absorbed dye at a PDMS ratio of 1:20 was significantly higher. Interestingly, the PDMS ratios of 1:10 and 1:15 also led to similar hardness values. The absorption depth, taken at the intersection of the extrapolated strong intensity decay with the baseline, was plotted as a function of hardness in Figure 6b. For all four PDMS compositions, the intensities that were far apart from the channel appeared to be very similar to the level obtained with undyed DI water, as can be seen in the graph in Figure 7g.

The remaining hydrophobic dye substance in the sealing layer was analyzed for the composition of PDMS 1:10 as shown in Figure 7. A comparison with Figure 7b, for which the microfluidic channel was still filled with the dyed fluid, shows that after rinsing the channel with 1 mL DI water, the absorbed rhodamine B remains in the sealing layer.

To compare with the microfluidic systems, which are completely made of PDMS and are widely used by many research groups, the channel was closed with a glass slide and coated with a 23-µm-thick PDMS layer. This simulated the full exposure to PDMS occurring in these systems. In Figure 7f, the intensity distribution in the channel is shown after introducing the dyed fluid and rinsing with DI water. First, the sidewall intensities were not more irregular, which can be explained by a less well-defined closure when compressing the two relatively soft components. Secondly, the remaining uptake of rhodamine B in the lid became visible after flushing the system, which indicated a problematic uptake of substances in systems made entirely of PDMS.

The LIF tests were repeated with membranes of PET and Si_3_N_4_ integrated between the sealing layer and the glass lid (Supplementary Figure S3). In all configurations, the chips were optically inspected for leakage, but no dyed fluid could be discovered outside the microchannel. However, the conclusiveness of this observation is limited, because a very thin fluid film would result in very weak fluorescence, which would be difficult to detect.

### 3.5. Leak Test

**Figure 8:**
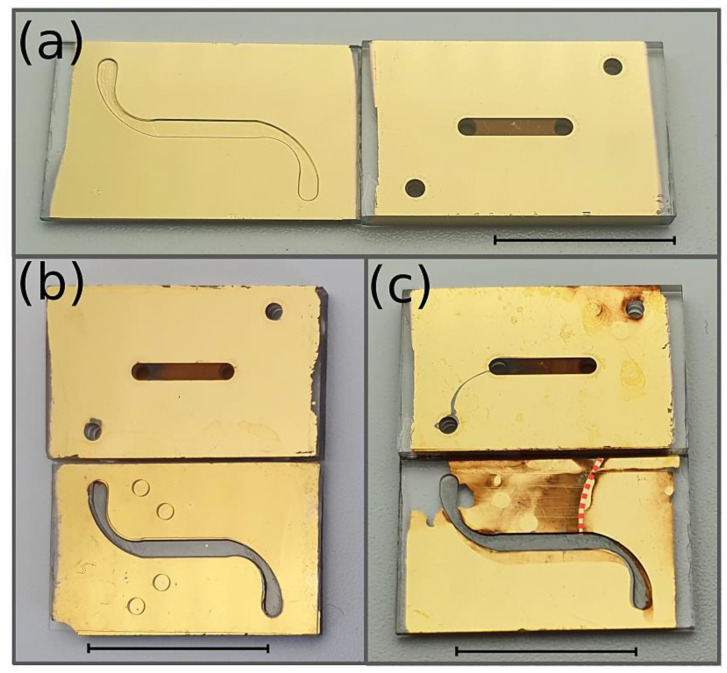
(**a**) The upper and lower section of the system coated with an intact gold layer before the leak test. (**b**) Microfluidic system using the shift method, after the leak test, using a potassium iodide (KI) solution. (**c**) Microfluidic system using the shift method with an obstacle (indicated by the red dashed line) to enforce leakage, after the leak test (negative control). Scale bar = 10 mm.

To detect even the smallest traces of leakage, the systems were tested using the self-developed gold etch leak test. Before the leak test, the surface had an intact gold coating, shown in Figure 8a. Utilizing the PDMS gasket, only the surface inside the microchannel was etched, as demonstrated in Figure 8b, while some etchant passed through the porous membrane into the lower channel and tarnished the gold layer. As in the control experiment, in Figure 8c, an artificial obstacle (represented by the red dashed line) was embedded in the sealing layer above the channel, causing a small gap in the sealing layer, which created leakage. This was made visible by the dark brown spots and the partly removed gold layer.

### 3.6. Transfer Method

**Figure 9:**
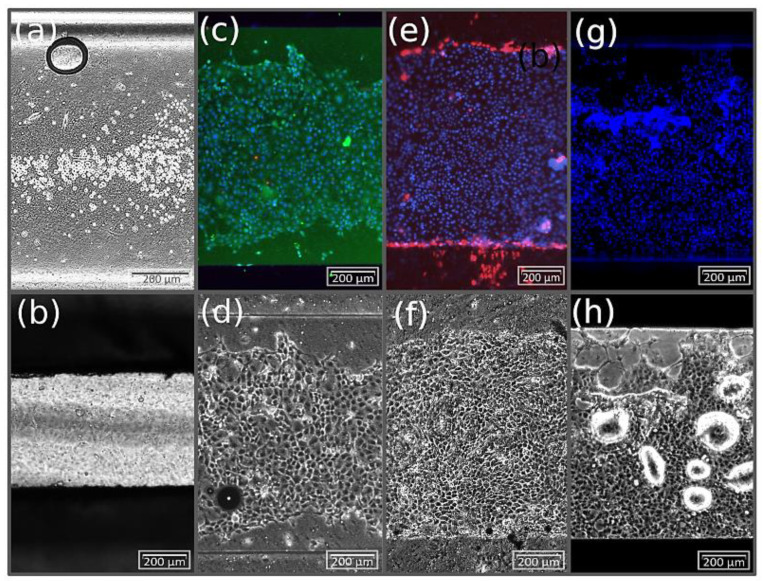
Comparison of on-chip MDCK cell distributions obtained with different seeding procedures. (**a**) Direct seeding by manual flow control and sedimentation inside the chip. (**b**) Cells after seeding for 12 h on a PET membrane in the transfer holder. Note that part of the image is shaded by the transfer holder. (**c**) Fluorescence image of live–dead staining and (**d**) bright-field images of the cell’s on PET membrane prior to the transfer into the chip. (**e**) Fluorescence image of the cell nucleus and dead staining and (**f**) bright-field image of cells on a PET membrane after on-chip cultivation. (**g**) Fluorescence image of cell nucleus staining and (**h**) a bright-field image of cells on a Si_3_N_4_ membrane prior to the transfer into the chip. In addition, small droplets underneath the highly transparent membrane became visible.

First, we investigated a direct seeding of the cells inside the closed chip, which is more typically practiced in organ-on-chip research. Following a multistep protocol for cleaning, coating, rinsing and seeding, all liquids had to be pushed through the connecting tubing using syringes. With this method, however, several difficulties became apparent. First, the cell distribution on the polymer membrane was inhomogeneous and air bubbles could easily enter the chip (Figure 9a). Second, the cells needed some time to sediment and to adhere. During this critical phase, the flow had to be stopped. Third, a relatively high fraction of cell suspension remained in the syringe and tubes, resulting in lower seeding densities. To obtain the same cell density on the membrane as in standard cell assays, more cells were necessary to compensate for the dead volumes in the tubing and syringes.

To demonstrate the advantages of the transfer method, MDCK cells were seeded into three transfer holders. Two holders were equipped with a collagen-coated cell culture membrane of PET and one holder was equipped with a collagen-coated membrane made of Si_3_N_4_.

In comparison to the direct injection method, the cell distribution became very homogenous. The cells quickly began to change their round morphology and spread.

First, adhered cells were observed on the membranes after approximately 3 h. After 6 h, this number increased significantly. A confluent cell layer with the typical cobblestone-like morphology of MDCK cells was detected after 12 h (Figure 9b). Before the transfer, the PET membrane of the first transfer holder was taken out for a fluorescence live–dead cell assay. As seen in Figure 9c from the calcein-AM staining, the number of vital cells is very high. In the bright-field image in Figure 9d, the PDMS sealing layer framing the cells beneath and above can be distinguished by the horizontal lines. The PET membrane of the second transfer holder was then transferred to the microfluidic chip as described above, using the shift method with a PDMS (1:10) sealing layer. Further cultivation was performed under dynamic flow conditions at a flow rate of 10 µL/min for 42 h. The proliferation of MDCK cells on the PET membrane was monitored throughout the cultivation period by video (Supplementary Video S3), which is supplied in the supplementary data. In this period, the cells colonized the available membrane area and started to condense, creating a confluent cell layer. In the beginning, the basic pressure in the upper channel was set to 90 mbar relative to the lower channel. This caused a delamination of cells closer to the inlet, which can be seen in the video of the passing cells.

Furthermore, by nonpermanent sealing, the transfer method enabled the possibility of characterizing cells outside the chip after dynamic cultivation. In particular, this facilitated complex processes such as immunocytochemical staining, both during performance and evaluation. As shown in Figure 9e, after cultivation, the membrane was taken out of the chip and placed in a 12-well plate for fluorescence microscopy. The staining assay was performed, using propidium iodide to stain dead cells (red) and Hoechst 33342 to counterstain the cell nuclei (blue). Due to the strong background signal of the calcein-AM, the green live staining was renounced. It could be observed that only a few dead cells were dispersed on the membrane. Most of them were found at the edges of the cell layer. This effect resulted from the disassembly of the chip system. As can be seen in the video, the MDCK cells strongly proliferated during cultivation, whereby they came into contact with the PDMS sealing layer. While opening the sealing layer, cells in direct contact with the layer were tore off. For this reason, staining with PI showed a concentration of dead cells at the edges of the membrane. However, in relation to the total growth area, the surface with damaged cells was very small. Overall, the cells developed a confluent, homogenous cell layer, as shown in Figure 9e,f.

Similar to the results on the PET membrane, the cells adhered and spread homogenously on the ultrathin membrane made of Si_3_N_4_, as can be seen in Figure 9g,h. Compared to the direct injection method, the transfer method offers high reproducibility, as the seeding process does not depend on the delicate handling of the injection procedure. Seeding with the transfer method can be performed under standardized conditions similar to the colonization process on Transwell^®^ inserts, requiring comparable practice and care in handling. We were able to perform the transfer method successfully in five experiments, which demonstrates the high reproducibility of this method.

### 3.7. TEER Measurement

**Figure 10:**
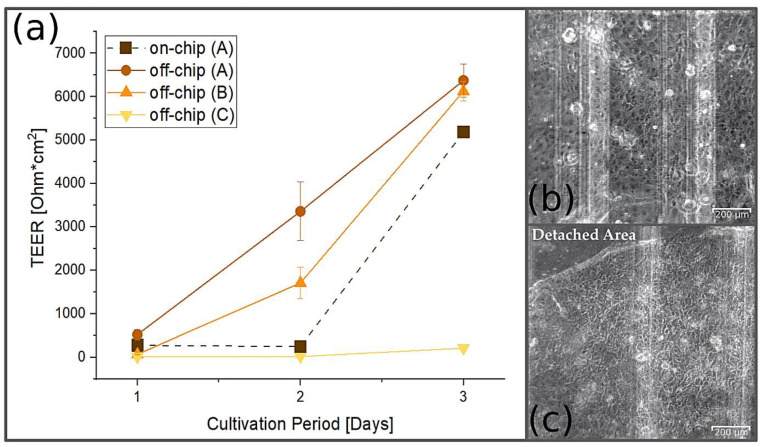
TEER values of MCDK cells obtained on-chip and off-chip at different proliferation stages. (**a**) After seeding with a density of 13,750 cells/mm^2^ on the transfer holder and various cultivation periods, the on-chip measurements were performed directly after transfer. Off-chip measurements were obtained in Transwell^®^ inserts (*n* = 3) with seeding densities of 13,750 cells/mm^2^ (A), 4500 cells/mm^2^ (B) and 900 cells/mm^2^ (C). (**b**) Brightfield image of MDCK cells inside the microfluidic chip with transparent interdigitated electrodes (their edges appear as vertical shadow lines) directly after the transfer, which took place one day after seeding. (**c**) Brightfield image of MDCK cells inside the microfluidic chip after progressed proliferation. With a higher confluence, the cell layer can detach and roll up more easily under uncareful handling, as shown in the upper left corner.

Figure 10a shows TEER values obtained with MDCK cells cultured off-chip in Transwell^®^ inserts and on-chip. Seeding densities of 13,750 cells/mm^2^, 4500 cells/mm^2^ and 900 cells/mm^2^ resulted after one day of cultivation at different stages of confluence. At this time and after further cultivation periods, measurements in the EndOhm chamber gave rather high TEER values for the higher seeding densities. The on-chip TEER values were measured using the integrated microelectrodes after the cells had been cultured in the transfer holder for one, two and three days and then transferred to the chip (see Figure 10b). It was observed that as the cells became more confluent in the transfer holder, by uncareful handling during transfer to the chip, they detached more easily from the polymer membrane and started to roll up (see Figure 10c). This created an area of low resistance and a drop in the measured TEER value. However, with experienced handling, these problems could be reduced and even avoided. Similar TEER values were obtained on-chip and off-chip. The small difference may still result from disturbances during the membrane transfer. The main result is that on-chip TEER measurements using transparent microelectrodes correlate very well with established conventional TEER measurements. This allows for the barrier quality to be monitored continuously in the chip without interrupting the dynamic perfusion, while simultaneous microscopic observation is possible.

## 4. Conclusions

We developed a new tissue barrier-on-chip system that allows the nonpermanent closure and sealing of organ-on-chip systems. The multilayered microfluidic chip is fabricated from inert glass, comprises embedded transparent microelectrodes for impedance measurements and can be closed with a very thin sealing interlayer of PDMS.

Despite the hydrophobic surface properties of PDMS, the utilization of only a very thin PDMS seal does not compromise the system’s inertness. Since we developed a unique scalable MEMS fabrication route that is suitable for organ-on-chip mass fabrication, the proof on concept was kept short. It was demonstrated that cells can be seeded externally on a membrane, similar to standard procedures such as using Transwell^®^ inserts. Through this process, very densely and homogeneously populated membranes can be transferred into the chip for further dynamic cell cultivation. The cell-populated membrane can be taken out of the chip at any time for detailed analysis such as fluorescence staining, histological cross-section cuts or for cryofixation and evaluation by SEM. In principle, the chip can be used many times for different experiments as well as for other types of tissue barriers. The nonpermanent closure allows for a free selection of membrane materials. The barrier tissue can be continuously monitored by TEER measurements using integrated transparent microelectrodes, as was demonstrated using MCDK cells.

Equipped with the features of tissue transfer capability, perfusion chamber inertness, gas trapping and integrated TEER monitoring, and supplemented with automated fluid control, the presented system is a promising platform to study established in vitro models of tissue barriers under reproducible microfluidic perfusion conditions. For the future parallelization of cell experiments, the microfluidic control will become more complex.

In this way, a realistic test practice under well-controlled conditions can be created to evaluate drug candidates in preclinical studies, thereby reducing animal experiments.

## Data Availability

The videos Video S1: Animation-Transfer, Video S2: MDCK: Transfer Air Bubbles, Video S3: MDCK-on-Chip has been made publicly available on https://zenodo.org/record/5825312.

## Supplementary Materials

The following supporting information can be downloaded at: https://www.mdpi.com/article/10.3390/pharmaceutics14071451/s1, Figure S1: MTT-cell viability test (a) Cell viability of MDCK cells after five days of exposure to the different materials used in the microfluidic experimental setup compared to the negative control (KRB Ref). Sample quantities are ranging *n* = 3–6 (adhesive tape only 3 material samples, in all other cases 6 material samples (2 runs with 3 samples had been tested) with error bars representing the SD (Standard Deviation). Figure S2. (a) Schematic view of the experimental setup for flow control showing the microsystem in the center of the retainer (with inlets I1 and I2, outlets O1 and O2, and side ports S1 and S2 and contact pad holes C1–4), culture medium reservoirs (M1, M2), waste reservoirs (W1, W2), pneumatic controllers (P1, P2, P3), the flow rate sensor (Q1) and sterile syringe filters connected to the side ports S1 and S2 which are closed during the experiments. The main setup is positioned on top of an inverse microscope placed in a self-built incubator (red frame) using a thermo-controller coupled to a panel heater and heating foils and to a Pt-100 temperature sensor. (b) Actual image of the entire experimental setup. (c) Photo of the microsystem in the center surrounded by the peripheral flow control. (d) Zoom in of the microsystem system with the chip placed in the microfluidic holder. Figure S3. Fluorescence images of the microfluidic upper channel with sealing layer and integrated membranes at 100 mbar. (a) Chip with PDMS sealing layer (1:10) and integrated PET-membrane filled with DI-water. (b) Chip with PDMS sealing layer (1:10) and integrated PET-membrane filled with rhodamin B. (c) Chip with PDMS sealing layer (1:10) and integrated Si3N4—membrane (dashed line) filled with rhodamin B. (d) Chip with PDMS sealing layer (1:10) and integrated Si3N4—membrane (dashed line) flushed with DI-water after it was filled with rhodamin B. Video S1: Animation-Transfer, Video S2: MDCK: Transfer Air Bubbles, Video S3: MDCK-on-Chip, https://zenodo.org/record/5825312.
