# Supplementary material for: Tissue Barrier-on-Chip: A Technology for Reproducible Practice in Drug Testing
Source: Pharmaceutics. 2022 Jul 12;14(7):1451. doi: 10.3390/pharmaceutics14071451 (PMC9323870; doi:10.3390/pharmaceutics14071451)
Supplement: Supplementary file 1 [file pharmaceutics-14-01451-s001.zip › pharmaceutics-1754465-supplementary.pdf]

Figure S1.

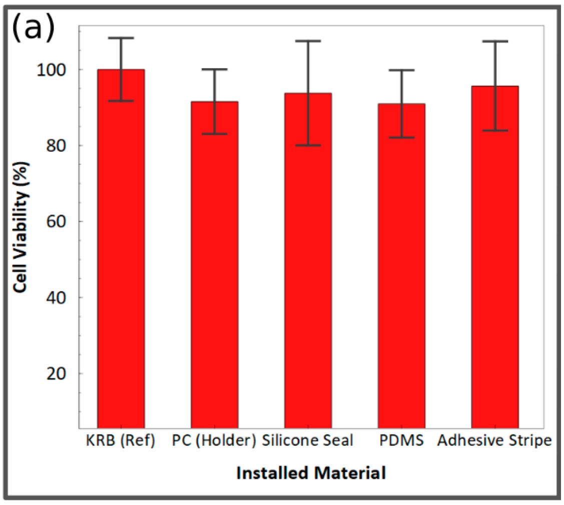

Figure S1. MTT-cell viability test (a) Cell viability of MDCK cells after five days of exposure to the different materials used in the microfluidic experimental setup compared to the negative control (KRB Ref). Sample quantities are ranging n=3-6 (adhesive tape only 3 material samples, in all other cases 6 material samples (2 runs with 3 samples had been tested) with error bars representing the SD (Standard Deviation).

Figure S2.

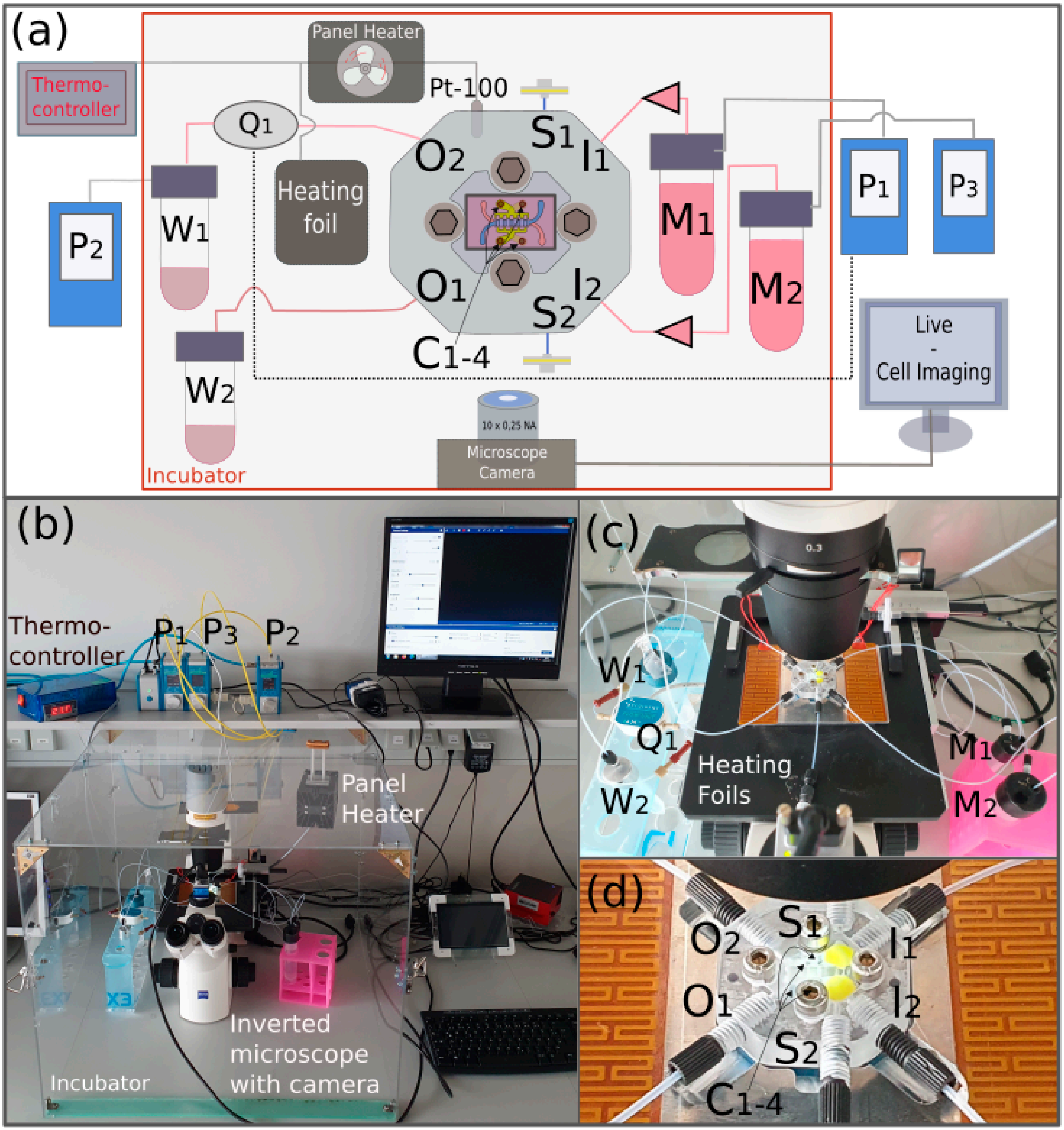

Figure S2. (a) Schematic view of the experimental setup for flow control showing the microsystem in the center of the retainer (with inlets I1 and I2, outlets O1 and O2, and side ports S1 and S2 and contact pad holes C1-4), culture medium reservoirs (M1, M2), waste reservoirs (W1, W2), pneumatic controllers (P1, P2, P3), the flow rate sensor (Q1) and sterile syringe filters connected to the side ports S1 and S2 which are closed during the experiments. The main setup is positioned on top of an inverse microscope placed in a self-built incubator (red frame) using a thermo-controller coupled to a panel heater and heating foils and to a Pt-100 temperature sensor. (b) Actual image of the entire experimental setup. (c) Photo of the microsystem in the center surrounded by the peripheral flow control. (d) Zoom in of the microsystem system with the chip placed in the microfluidic holder .

Figure S3.

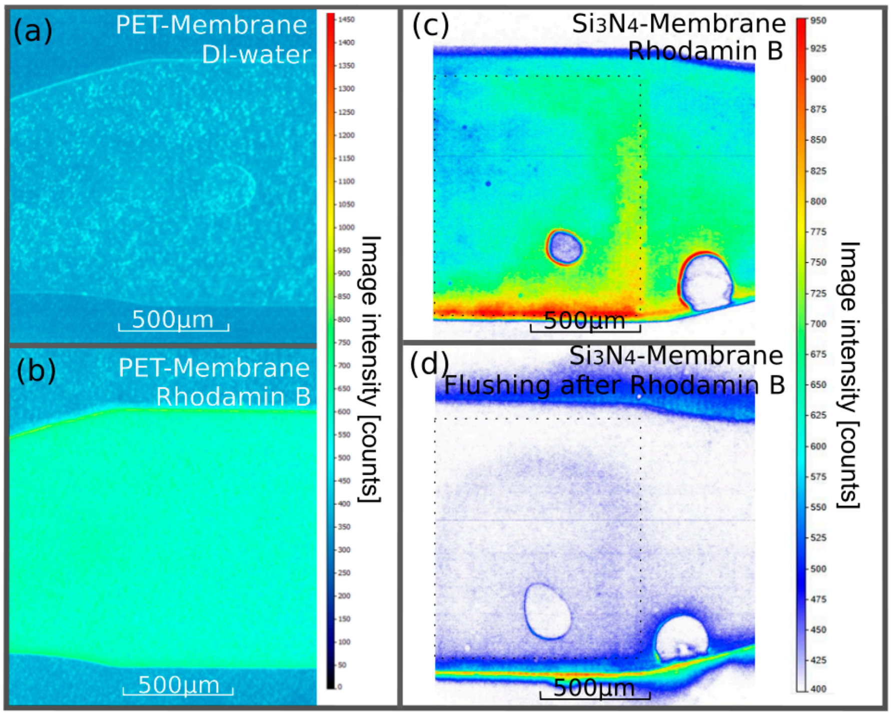

Figure S3. Fluorescence images of the microfluidic upper channel with sealing layer and integrated membranes at 100 mbar. (a) Chip with PDMS sealing layer (1:10) and integrated PET-membrane filled with DI-water. (b) Chip with PDMS sealing layer (1:10) and integrated PET-membrane filled with rhodamin B. (c) Chip with PDMS sealing layer (1:10) and integrated Si3N4—membrane (dashed line) filled with rhodamin B. (d) Chip with PDMS sealing layer (1:10) and integrated Si3N4--membrane (dashed line) flushed with DI-water after it was filled with rhodamin B.
